# Embryological and pregnancy outcomes of IVF-ET in overweight/obese and normal-weight women with PCOS

**DOI:** 10.3389/fendo.2025.1552561

**Published:** 2025-03-27

**Authors:** Shuo Huang, Zheng Wang, Rui Yang, Rong Li, Jie Qiao

**Affiliations:** ^1^ State Key Laboratory of Female Fertility Promotion, Center for Reproductive Medicine, Department of Obstetrics and Gynecology, Peking University Third Hospital, Beijing, China; ^2^ National Clinical Research Center for Obstetrics and Gynecology, Peking University Third Hospital, Beijing, China; ^3^ Key Laboratory of Assisted Reproduction (Peking University), Ministry of Education, Beijing, China; ^4^ Beijing Key Laboratory of Reproductive Endocrinology and Assisted Reproductive Technology, Peking University Third Hospital, Beijing, China; ^5^ Center for Reproductive Medicine, Department of Obstetrics and Gynecology, Peking University Third Hospital, Beijing, China

**Keywords:** PCOS, IVF-ET, obesity, live birth, infertility

## Abstract

**Introduction:**

Female obesity has been conclusively associated with compromised fertility, adverse pregnancy outcomes and higher risks of obstetric and neonatal complications. However, it remains unclear whether the adverse outcomes observed in IVF treatments among women with obesity are primarily due to obesity itself or to underlying pathologies such as PCOS. Studies investigating the impact of overweight/obesity compared to normal weight in women with PCOS have yielded inconsistent findings.

**Methods:**

We retrospectively analyzed 4083 women with PCOS undergoing the first IVF-ET cycle with antagonist protocol. Among them, 1755 were divided into the normal weight group (18.5 g/m^2^ ≤ BMI < 24.0 kg/m^2^), 1398 into the overweight group (24.0 kg/m^2^ ≤ BMI < 28.0 kg/m^2^) and 930 into the obese group (BMI ≥ 28.0 kg/m^2^). The primary outcome was live birth. Other outcomes were cycle parameters, embryological, pregnancy outcomes and birth weight of newborns. We additionally investigated potential associations of maternal BMI as a continuous variable with outcomes for both linear associations and non-linear associations.

**Result:**

Women with overweight and obese had fewer numbers of oocytes retrieved (adjusted B: -0.82 [-1.17 to -0.47] and adjusted B: -1.86 [-2.26 to -1.46], respectively), numbers of 2PN (adjusted B: -0.52 [-0.78 to -0.26] and adjusted B: -1.86 [-2.26 to -1.46]), and numbers of good-quality embryos (adjusted B: -0.34 [-0.57 to -0.12] and adjusted B: -0.88 [-1.13 to -0.62]), compared to the women with normal weight. The live birth rate was 35.7%, 30.6% and 27.2% in the normal weight group, the overweight group and obese group, respectively (adjusted OR:0.76 [0.65 to 0.89]) for overweight verse normal weight, and adjusted OR:0.64 [0.53 to 0.76)] for obese verse normal weight). There were significant associations between higher BMI and adverse outcomes. We did not observe significant non-linear associations between BMI and these outcomes.

**Discussion:**

Overweight or obese women with PCOS undergoing IVF-ET experienced lower numbers of oocytes and good quality embryos, reduced rates of live births, and higher rates of miscarriage compared to normal-weight women with PCOS.

## Introduction

Polycystic ovary syndrome (PCOS), the most common endocrine disorder in women of reproductive age, affects 10–13% of women (1). In China, a recent epidemiological study found that the incidence of PCOS is 7.8% (2). PCOS is the leading cause of dysmetabolic infertility, characterized by chronic anovulation, hyperandrogenemia, and the presence of polycystic ovaries. Excess androgens play a central role in the condition, promoting the formation of primordial, primary, secondary, and early preantral follicles while inhibiting their progression to the dominant follicle stage due to impaired granulosa cell function (3, 4). This disruption in follicular development ultimately leads to anovulation and infertility (3, 4).Obesity is closely associated with PCOS and up to 60% of women with PCOS are overweight or obese (5). Female obesity has been conclusively associated with compromised fertility, adverse pregnancy outcomes and higher risks of obstetric and neonatal complications (6, 7). In infertile women undergoing *in vitro* fertilization embryo transfer (IVF-ET) treatment, research indicated that those with obesity had poorer clinical outcomes compared to women with normal weight, such as the need for higher doses of gonadotrophins, elevated cycle cancellation rate, increased miscarriage rate, and lower live birth rate (8).

Although the association between obesity and poor reproductive outcomes in IVF-ET is relatively clear (9), considering the bidirectional relationship between obesity and PCOS, where obesity promotes the occurrence and development of PCOS, and PCOS in turn leads to weight gain (10), it remains unclear what impact PCOS has on poor reproductive outcomes in women with obesity. The existing studies are heterogeneous in terms of population, baseline characteristics, IVF methods and outcomes explored, which prevents drawing firm conclusions (11–14). For instance, clinical characteristics of PCOS and infertility often exhibit differences based on ethnicity (15). Many studies have indicated that women with PCOS and obesity experienced poorer pregnancy outcomes compared to their counterparts with normal weight, as classified by international BMI cutoffs (12, 13). However, a study from China in 2018 yielded contradictory results, suggesting that high body mass index (BMI) had no adverse effects on IVF outcomes (16).

Thus, there is controversy regarding the outcomes of IVF-ET among PCOS women with different BMIs when categorized according to Chinese weight standards (17). Accurate information is crucial for clinicians as it guides IVF treatment strategies for women with PCOS. This study aimed to evaluate and compare the clinical outcomes following IVF-ET among women with PCOS who were categorized as normal weight, overweight, and obese.

## Method

### Subjects

We performed a retrospective cohort study in the Center of Reproductive Medicine of Peking University Third Hospital. The clinical data were retrieved from the clinical database. The study was approved by the local ethics committee (No. IRB00006761-M2024558). We included women diagnosed with PCOS and underwent the first IVF-ET with antagonist protocol between January 1, 2015, and December 31, 2022. The diagnosis of PCOS was based on the Rotterdam criteria (18). The exclusion criteria were (1) women with medical conditions or diseases contraindicated for pregnancy or recurrent miscarriage; (2) ongoing weight loss therapy or any concomitant treatment or history of gastrointestinal surgery; (3) endocrine disorders, including diabetes mellitus or thyroid disorders (hyperthyroidism, hypothyroidism); (4) uterine anomalies (e.g., untreated submucosal fibroids, adhesions, malformations) or untreated tubal hydrosalpinx affecting assisted conception outcomes; (5) couples with oocyte or sperm donation, *in vitro* maturation (IVM), preimplantation genetic testing (PGT) cycles. BMI was defined as weight (kilograms) divided by the square of height (meters). Based on the recommended Chinese BMI cut-off points (17), women were divided into three groups: normal weight (18.5 g/m^2^≤BMI<24.0 kg/m^2^), overweight (24.0 kg/m^2^≤ BMI<28.0 kg/m^2^), and obese (BMI≥28.0 kg/m^2^).

### Controlled ovarian hyperstimulation and embryo transfer protocol

All patients underwent ovarian stimulation with a standard antagonist protocol. The protocol consisted of daily stimulation with gonadotropin from day 2 of the menstrual cycle. Doses were selected by treating physician’s discretion. Gonadotropin releasing hormone antagonist (GnRH-ant) cetrorelix acetate (Cetrotide; Merck Serono) in a dose of 0.25 mg was administered when the dominant follicle measured 14 mm in diameter. When at least two dominant follicles were measured ≥17 mm in diameter, 250 µg of recombinant human chorionic gonadotropin (Ovidrel; Merck Serono) was administered for triggering. Transvaginal ultrasound-guided oocyte retrieval was performed 36 h after triggering. Oocytes were fertilized by conventional IVF or intracytoplasmic sperm injection (ICSI) according to sperm situation of male partner. Embryo culture and transfer procedures were performed according to the standard protocols of our center. Fresh embryo transfers occurred on either day 3 or day 5 after oocyte retrieval. The selection of the embryo transfer strategy is a collaborative decision involving both physicians and patients, tailored to individual circumstances and medical histories. In unselected populations, the preferred transfer strategy is either double embryo transfer (DET) at cleavage stage or single embryo transfer (SET) at blastocyst stage. Luteal support protocol is progesterone vaginal gel (Crinone 8%, Merck Serono) 90mg daily from the day of oocyte retrieval.

### Outcomes

Our primary outcome was live birth. Other outcomes were cycle parameters, embryological and pregnancy outcomes. A clinical pregnancy was defined after sonographic evidence of an intrauterine gestational sac was observed. A live birth was defined as the birth of an infant after 28 weeks gestation with postnatal evidence of life. Pregnancy loss included ectopic pregnancy, miscarriage, stillbirth and termination of pregnancy.

### Statistical analysis

Baseline characteristics were presented differently depending on the type of variable: continuous variables with a normal distribution were expressed as mean and standard deviation (SD), skewed variables as median and interquartile range (IQR), and dichotomous variables as percentages. The comparison among the three groups was conducted using ANOVA test for normally distributed variables, Kruskal–Wallis test for non-normally distributed variables, and Chi-square tests for dichotomous variables. Further intergroup comparisons were made using the Bonferroni *post-hoc* test.

For outcome variables, the presentation followed the same format as baseline characteristics, and comparisons between groups were performed using linear regression or logistic regression. Regression models were fitted to calculate B coefficients or odds ratios (OR) along with their 95% confidence intervals (CIs), with the normal weight group serving as the reference. Adjusted effect sizes were obtained by incorporating female age, primary infertility, and antral follicle count (AFC) into the regression models.

We additionally investigated potential associations of maternal BMI as a continuous variable with embryonic and pregnancy outcomes for both linear associations by logistic regression or linear regression where relevant and non-linear associations by restricted cubic spline regression with three knots (25^th^: 22.0 kg/m^2^, 50^th^: 24.8 kg/m^2^, and 75^th^: 27.5 kg/m^2^ percentiles).

A P-value of <0.05 was considered to indicate statistical significance. Statistical analysis was performed in SPSS 25.0 (IBM, Chicago, IL, USA) and Stata 17.0 (Statacorp, College Station, TX, USA).

## Results

From January 2015 to December 2022, a total of 4083 women with PCOS undergoing the first IVF-ET cycle were included in this study. Among them, 1755 were divided into the normal weight group, 1398 into the overweight group and 930 into the obese group.

Baseline characteristics including age, duration of infertility, proportion of primary infertility, and basal hormone levels are presented in **Table 1**. Women in the obese group were younger, had a longer duration of infertility, and a higher proportion of primary infertility compared to both the overweight group and the normal weight group. Additionally, they exhibited lower basal PRL levels and higher AFC than both the overweight group and the normal weight group. LH/FSH ratio and basal E2 levels did not differ among three groups.

**Table 1:** Characteristics of the normal weight, overweight and obese women with PCOS who underwent IVF-ET.

	Normal weight N=1755	Overweight N=1398	Obese N=930	P value
Age (years)	31.2±4.5^b^	31.3±4.5^c^	30.7±4.0^b,c^	<0.001
BMI (kg/m^2^)	21.6±1.5^a,b^	25.8±1.1^a,c^	30.5±2.1^b,c^	<0.001
Duration of infertility (years)	3.8±3.0^a,b^	4.1±3.0^a,c^	4.5±2.9^b,c^	<0.001
Primary infertility	1173 (66.8%)^b^	908 (64.9%)^c^	650 (69.9%)^b,c^	0.046
Cause of infertility				
PCOS only	565 (32.2%)	439 (31.4%)	305 (32.8%)	0.77
PCOS and tubal factor	459 (26.2%)	357 (25.5%)	256 (27.5%)	0.56
PCOS and male factor	828 (47.2%)	700 (50.1%)	448 (48.2%)	0.27
PCOS and others	220 (12.5%)^b^	159 (11.4%)^c^	89 (9.6%)^b,c^	0.07
Basal LH (mIU/ml)	6.2±4.7	5.9±5.4	5.7±4.0	0.12
Basal FSH (mIU/ml)	6.8±2.7^a,b^	6.3±2.2^a^	6.3±2.3^b^	<0.001
LH/FSH ratio	1.0±1.4	1.0±0.8	1.0±0.7	0.54
Basal E2 (pmol/L)	163 (128; 206)	162 (127; 206)	169 (129; 218)	0.17
Basal PRL (ng/ml)	12.1 (8.6; 17.4)^a,b^	11.2 (7.8; 15.1)^a,c^	10.4 (7.7; 13.6)^b,c^	<0.001
Basal P (nmol/L)	1.1 (0.9; 1.5)^b^	1.1 (0.8; 1.5)^c^	1.0 (0.7; 1.4)^b,c^	<0.001
Basal T (nmol/L)	0.7 (0.7, 1.0)^a,b^	0.8 (0.7, 1.2)^a^	0.8 (0.7, 1.2)^b^	<0.001
Basal A (nmol/L)	8.8±4.5^a^	9.9±5.0^a^	9.5±4.7	<0.001
AMH (ng/ml)	6.6±4.4^b^	6.2±3.9	5.7±3.7^b^	<0.001
AFC	15.7±7.0^a,b^	17.0±6.8^a,c^	18.0±6.7^b,c^	<0.001

Values are presented as mean±SD or median (IQR) or n (%).

Superscript letters indicate between-group differences. a indicates a significant difference between normal weight and overweight group; b indicates a significant difference between normal weight and obese group; and c indicates a significant difference between overweight and obese group.

BMI, body mass index; PCOS, polycystic ovary syndrome; LH, luteinizing hormone; FSH, follicle-stimulating hormone; E2, estradiol; PRL, prolactin; P, progesterone; T, testosterone; A, androstenedione; AFC, antral follicle count; AMH, anti-müllerian hormone.

As for ovarian response parameters and embryological outcomes (**Table 2**), we observed longer stimulation days (adjusted B: -0.82 [-1.17 to -0.47] for overweight versus normal weight and adjusted B: -1.86 [-2.26 to -1.46] for obese versus normal weight, respectively) and higher consumption of gonadotrophins (adjusted B: -0.82 [-1.17 to -0.47] for overweight versus normal weight and adjusted B: -1.86 [-2.26 to -1.46] for obese versus normal weight, respectively). Moreover, women with overweight and obese had fewer numbers of oocytes retrieved (adjusted B: -0.82 [-1.17 to -0.47]and adjusted B: -1.86 [-2.26 to -1.46], respectively), numbers of 2PN (adjusted B: -0.52 [-0.78 to -0.26] and adjusted B: -1.86 [-2.26 to -1.46]), and number of good-quality embryos (adjusted B: -0.34 [-0.57 to -0.12]and adjusted B: -0.88 [-1.13 to -0.62]), compared to the women with normal weight. More than 95% women had two cleavage-stage embryos transferred in three groups, with a significant difference between the overweight group and the normal weight group (adjusted OR: 1.50 [1.01 to 2.23]) but not in the obese group and the normal weight group (adjusted OR: 1.20 [0.78 to 1.84]).

**Table 2:** Ovarian response parameters and embryological outcomes in the normal weight, overweight and obese women with PCOS.

	Normal weight N=1755	Overweight N=1398	Obese N=930
Stimulation days	10.2±1.9	10.7±2.3	11.4±2.3
Crude B (95%CI)	REF	**0.5 (0.4 to 0.7)**	**1.2 (1.0 to 1.4)**
Adjusted B (95%CI)	REF	**0.5 (0.4 to 0.7)**	**1.1 (1.0 to 1.3)**
Doses of gonadotropins (IU)	1815.5±813.2	2024.1±843.9	2352.2±909.4
Crude B (95%CI)	REF	**209 (149 to 268)**	**537 (469 to 604)**
Adjusted B (95%CI)	REF	**254 (196 to 312)**	**633 (567 to 699)**
E2 on trigger day (pmol/L)	8470.3±4497.3	7513.4±4184.4	7118.0±4383.9
Crude B (95%CI)	REF	**-957 (-1267 to 646)**	**-1352 (-1704 to -1000)**
Adjusted B (95%CI)	REF	**-1122 (-1440 to -803)**	**-1682 (-2043 to -1321)**
Endometrial thickness (mm)	10.4±1.7	10.4±1.7	10.5±1.8
Crude B (95%CI)	REF	-0.04 (-0.18 to 0.09)	0.02 (-0.14 to 0.17)
Adjusted B (95%CI)	REF	-0.04 (-0.18 to 0.10)	0.01 (-0.16 to 0.17)
Number of retrieved oocyte	10.9±5.1	10.2±5.0	9.5±4.7
Crude B (95%CI)	REF	**-0.61 (-0.96 to -0.26)**	**-1.39 (-1.79 to -0.99)**
Adjusted B (95%CI)	REF	**-0.82 (-1.17 to -0.47)**	**-1.86 (-2.26 to -1.46)**
Number of 2PN	6.5±3.7	6.1±3.6	5.5±3.4
Crude B (95%CI)	REF	**-0.41 (-0.66 to -0.16)**	**-1.01 (-1.30 to -0.73)**
Adjusted B (95%CI)	REF	**-0.52 (-0.78 to -0.26)**	**-1.26 (-1.56 to -0.97)**
Number of good-quality embryo	4.8±3.3	4.5±3.1	4.1±2.9
Crude B (95%CI)	REF	**-0.28 (-0.50 to -0.06)**	**-0.68 (-0.93 to -0.43)**
Adjusted B (95%CI)	REF	**-0.34 (-0.57 to -0.12)**	**-0.88 (-1.13 to -0.62)**
Number of transferred embryo	1.9 ±0.3	1.9 ±0.3	1.8±0.4
Crude B (95%CI)	REF	0.002 (-0.02 to 0.03)	**-0.03 (-0.06 to -0.002)**
Adjusted B (95%CI)	REF	0.002 (-0.02 to 0.03)	**-0.05 (-0.07 to -0.02)**
Transferred with cleavage-stage embryos	1677 (95.6%)	1353 (96.8%)	894 (96.1%)
Crude OR (95%CI)	REF	1.40 (0.96 to 2.03)	1.16 (0.77 to 1.73)
Adjusted OR (95%CI)	REF	**1.50 (1.01 to 2.23)**	1.20 (0.78 to 1.84)

Values are presented as mean±SD or n (%).

E2, estradiol; PN, pronucleus; REF, reference.

Bold numbers indicate p-value < 0.05.

Data were adjusted for age, primary infertility and AFC.

Fertility outcomes of IVF-ET are presented in **Table 3**. The live birth rate was 35.7%, 30.6% and 27.2%in the normal weight group, the overweight group and obese group, respectively. Compared to women with normal weight, women with overweight had significantly lower live birth rate (adjusted OR:0.76 [0.65 to 0.89]), as well as women with obesity (adjusted OR:0.64 [0.53 to 0.76)]). Obese women with PCOS had a 36% lower likelihood of achieving live birth compared to normal weight women with PCOS. Early miscarriage rate was 11.1%, 13.6%, and 17.4% and late miscarriage rate was 2.9%, 5.8%, and 10% in the normal weight, overweight, and obese group, respectively. Women in the obese group had significantly higher early miscarriage rate (adjusted OR: 1.76 [1.21 to 2.56] and late miscarriage rate (adjusted OR: 3.93 [2.20 to 7.02]) compared to women with normal weight. Rates for twin pregnancy and ectopic pregnancy did not differ between the obese group and the normal weight group or between the overweight group and the normal weight group. There were significantly more premature births in the obese group than the normal weight group (adjusted OR: 1.64 [1.14 to 2.38]). There was no significant difference in the incidence of premature births between the overweight group and the normal weight group (adjusted OR: 1.16 [0.83 to 1.63]). The neonatal birthweight of singletons or twins did not differ significantly between the groups, with similar mean values observed across all groups.

**Table 3:** Fertility outcomes of IVF-ET in the normal weight, overweight and obese women with PCOS.

	Normal weight N=1755	Overweight N=1398	Obese N=930
Clinical pregnancy	731/1755 (41.7%)	538/1398 (38.5%)	350/930 (37.6%)
Crude OR (95%CI)	REF	0.88 (0.76 to 1.01)	**0.85 (0.72 to 0.995)**
Adjusted OR (95%CI)	REF	**0.84 (0.72 to 0.97)**	**0.81 (0.68 to 0.96)**
Live birth	626/1755 (35.7%)	428/1398 (30.6%)	253/930 (27.2%)
Crude OR (95%CI)	REF	**0.80 (0.69 to 0.92)**	**0.67 (0.57 to 0.80)**
Adjusted OR (95%CI)	REF	**0.76 (0.65 to 0.89)**	**0.64 (0.53 to 0.76)**
Early miscarriage	81/731 (11.1%)	73/538 (13.6%)	61/350 (17.4%)
Crude OR (95%CI)	REF	1.26 (0.90 to 1.77)	**1.69 (1.18 to 2.43)**
Adjusted OR (95%CI)	REF	1.26 (0.88 to 1.80)	**1.76 (1.21 to 2.56)**
Late miscarriage	21/731 (2.9%)	31/538 (5.8%)	35/350 (10%)
Crude OR (95%CI)	REF	**2.07 (1.17 to 3.64)**	**3.76 (2.15 to 5.56)**
Adjusted OR (95%CI)	REF	**2.29 (1.27 to 4.12)**	**3.93 (2.20 to 7.02)**
Termination of pregnancy with deformity	3/731 (0.4%)	4/538 (0.7%)	1/350 (0.3%)
Crude OR (95%CI)	REF	1.82 (0.41 to 8.16)	0.70 (0.07 to 6.71)
Adjusted OR (95%CI)	REF	1.79 (0.39 to 8.12)	0.64 (0.07 to 6,21)
Twin pregnancy	241/731 (33.0%)	179/538 (33.3%)	130/350 (37.1%)
Crude OR (95%CI)	REF	1.01 (0.80 to 1.28)	1.20 (0.92 to 1.57)
Adjusted OR (95%CI)	REF	1.07 (0.83 to 1.37)	1.18 (0.90 to 1.56)
Ectopic pregnancy	44/1755 (2.5%)	39/1398 (2.8%)	15/930 (1.6%)
Crude OR (95%CI)	REF	1.12 (0.72 to 1.73)	0.64 (0.35 to 1.15)
Adjusted OR (95%CI)	REF	1.20 (0.75 to 1.90)	0.61 (0.33 to 1.16)
Still birth	0	2	0
Premature birth	104/626 (16.6%)	81/428 (18.9%)	64/253 (25.3%)
Crude OR (95%CI)	REF	1.20 (0.86 to 1.63)	**1.72 (1.21 to 2.45)**
Adjusted OR (95%CI)	REF	1.16 (0.83 to 1.63)	**1.64 (1.14 to 2.38)**
birthweight of singletons (g)	3227±508.8	3294±591.5	3257±690.4
Crude B (95%CI)	REF	66.4 (-16.1 to 148.9)	30.0 (-71.4 to 131.4)
Adjusted B (95%CI)	REF	75.4 (-9.3 to 160.1)	32.2 (-69.5 to 134)
birthweight of twins (means, g)	2478±526.3	2524±433.7	2466±520.7
Crude B (95%CI)	REF	46.1 (-77.6 to 169.7)	-12.3 (-147.6 to 123.0)
Adjusted B (95%CI)	REF	72.8 (-53.5 to 199)	7.8 (-133 to 149)

Values are presented as mean±SD or n (%).

REF, reference; NA, not applicable.

Bold numbers indicate p-value < 0.05.

Data were adjusted for age, primary infertility and AFC.

**Figure 1:**
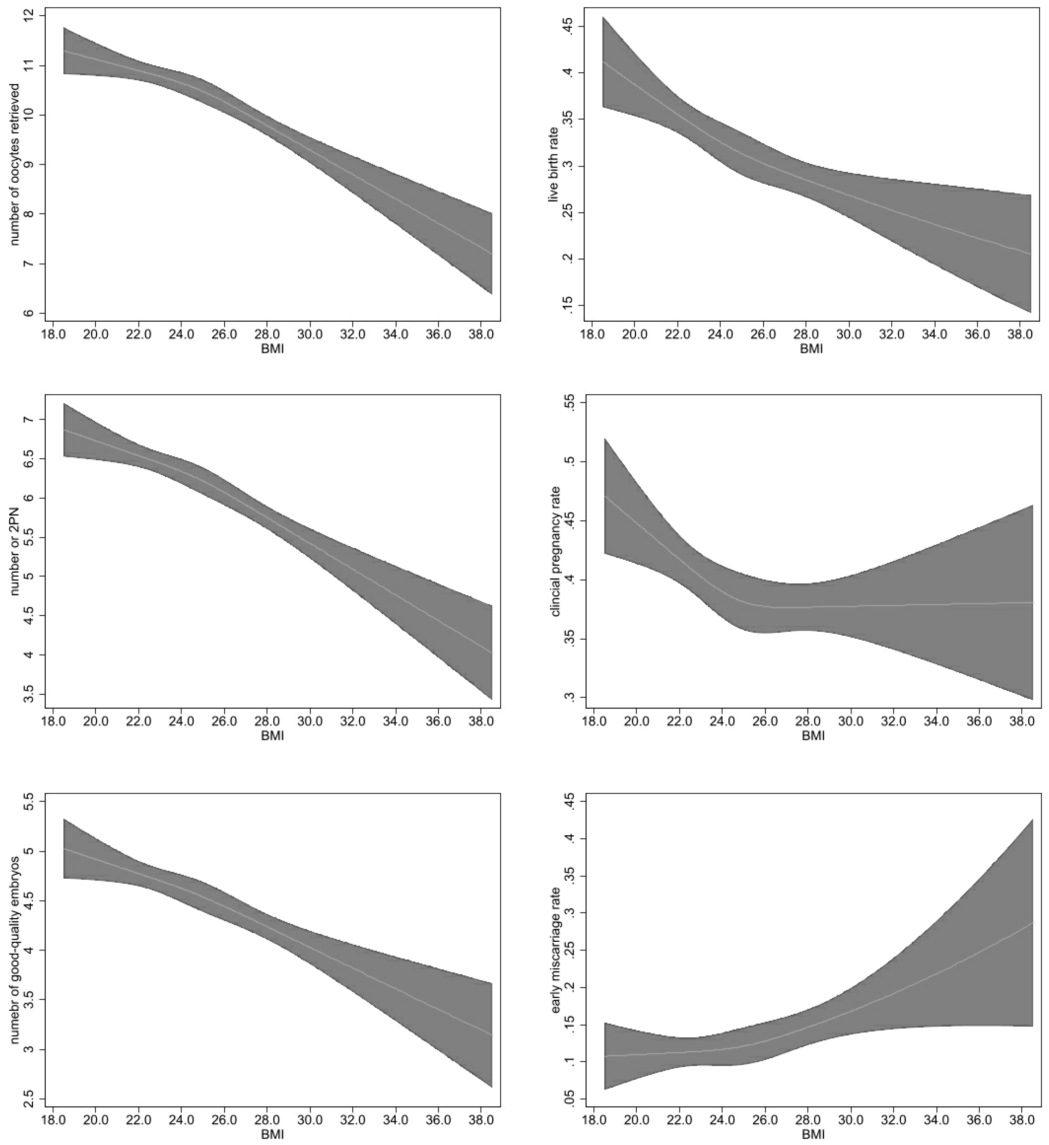
Non-linear associations between BMI and embryological and pregnancy outcomes The figure shows restricted cubic splines of embryological and pregnancy outcomes and BMI. The regression line for each spline segment for BMI levels is connected with smoothed transitions. The gray area represents the 95% CI. Data were adjusted for age, primary infertility and AFC.

BMI was also analyzed as a continuous variable to explore potential associations with embryonic and pregnancy outcomes, considering both linear (**Table 4**) and non-linear (**Figure 1**) associations. Both unadjusted and adjusted models revealed significant effects of BMI on various outcomes. Adjusted models showed a decrease in the number of retrieved oocytes (adjusted B: -0.20 [-0.24 to -0.15]), the number of 2PN (adjusted B: -0.14 [-0.17 to -0.11]), and the number of good-quality embryos (adjusted B: -0.09 [-0.12 to -0.07]). There were decreases in the likelihood of clinical pregnancy (adjusted OR: 0.98 [0.96 to 0.99]), live birth (adjusted OR: 0.95 [0.93 to 0.97]), and increases in the odds of early miscarriage (adjusted OR: 1.06 [1.02 to 1.10]). No non-linear associations of BMI with these outcomes were observed, and restricted cubic spline regression did not reveal any significant deviations from linear associations. Graphs illustrating these findings are presented in **Figure 1**.

**Table 4:** Linear associations of embryological and pregnancy outcomes with maternal BMI analyzed as a continuous variable.

Variable	Crude OR or B (95% CI)	p-value	Adjusted OR or B (95% CI)	p-value
Number of retrieved oocyte	-0.15 (-0.19 to -0.10)	<0.001	-0.20 (-0.24 to -0.15)	<0.001
Number of 2PN	-0.11 (-0.14 to -0.08)	<0.001	-0.14 (-0.17 to -0.11)	<0.001
Number of good-quality embryo	-0.07 (-0.10 to 0.05)	<0.001	-0.09 (-0.12 to -0.07)	<0.001
Clinical pregnancy	0.98 (0.97 to 0.999)	0.03	0.98 ((0.96 to 0.99)	0.007
Live birth	0.96 (0.94 to 0.97)	<0.001	0.95 (0.93 to 0.97)	<0.001
Early miscarriage	1.06 (1.02 to 1.10)	0.002	1.06 (1.02 to 1.10)	0.002

Data were adjusted for age, primary infertility and AFC.

## Discussion

In this study, we investigated the embryological and clinical outcomes following IVF-ET among women with PCOS who were categorized by their weight status: normal weight, overweight, and obese. Our findings revealed that overweight or obese women with PCOS undergoing IVF-ET experienced lower numbers of oocytes and good quality embryos, reduced rates of live births, and higher rates of miscarriage compared to normal-weight women with PCOS. We also analyzed the impact of BMI as a continuous variable on these outcomes, observing significant associations between higher BMI and adverse outcomes. Additionally. we did not observe significant non-linear associations between BMI and these outcomes.

The significant association between obesity and infertility has led an increasing number of women with obesity to seek infertility treatments like IVF (19). However, these treatments yielded lower success rates among them compared to normal-weight women. Clinical research has revealed that women with obesity experience impaired response to ovarian stimulation (20) and diminished success rates in IVF procedures (8, 21). Several factors contribute to this reduced efficacy, including suboptimal response of gonadotropin, compromised quality of oocytes/embryos, impaired uterine receptivity, and increased risk of miscarriage (22–25). However, it remains unclear whether the adverse outcomes observed in IVF treatments among women with obesity are primarily due to obesity itself or to underlying pathologies such as PCOS. Most research subjects are from the general obese population. Therefore, reducing heterogeneity and studying the impact of BMI on IVF outcomes in the PCOS population alone would be meaningful. Notably, it has been reported that in women with PCOS, obesity had significant negative effects on live birth rates (13, 26). On the other hand, there is one study reported that BMI was not associated with IVF outcomes in women with PCOS (16).

In our study with a large sample size, overweight or obese women with PCOS undergoing IVF-ET experienced lower quality oocytes/embryos and reduced rates of live births compared to normal-weight women with PCOS. These findings are in line with previous research (11, 26). Thus, we assume that obesity is a risk factor for embryological and pregnancy outcomes independent of PCOS. Our regression analysis, utilizing BMI as a continuous variable, further supports this conclusion, revealing a significant association between increased BMI and inferior outcomes.

Moreover, we find that obesity increases the risk of early miscarriage around two-fold. This finding aligns with a previous study conducted with a Chinese population, despite the application of different standards to define obesity (26). In addition, our findings indicated that obesity was associated with an almost four-fold increase in the rate of late miscarriage compared to the normal weight group. Data regarding the association of obesity and late miscarriage in women with PCOS is limited. Given the low incidence of late miscarriage, the confidence interval was wide. Further research is necessary to fully explore the exact effect of obesity on late miscarriage in women with PCOS.

The rate of premature birth was significantly elevated in the obese group compared to the normal weight group. Previous research has explored the association between maternal obesity and the risk of preterm delivery in a cohort of women without known PCOS status, revealing that maternal overweight and obesity during pregnancy were linked to heightened risks of preterm delivery, particularly extremely preterm delivery (27). Obesity can disrupt placental function, potentially leading to preterm birth (28). Thus, it is important to provide women with obesity and PCOS with relevant information regarding the health of their offspring. Additionally, in our cohort, we did not observe any significant differences in neonatal birth weight.

Exploring whether PCOS women with obesity have adverse outcomes in IVF treatment compared with normal-weight women is important, since it could provide further clinical guidance with respect to what kind of treatment might be needed for these women. Our current analysis suggests that to improve oocyte/embryo quality and increase the live birth rate, addressing obesity through interventions such as weight loss in women with PCOS before undergoing IVF treatment may be necessary.

The strength of our study lies in the large sample size, which surpasses that of many other investigations into the influence of BMI on IVF outcomes in women with PCOS. Departing from the WHO standardized classification of BMI 25 and 30, we opted for BMI 24 and 28 as cutoff points, aligning with guidelines for overweight and obesity in Chinese adults. In addition, we explored both linear and non-linear associations between BMI and outcomes. No non-linear associations were observed, contributing novel insights to the existing research.

However, our study has several limitations. One notable limitation is its retrospective nature, which may have introduced bias into the analysis. There were significant differences in age among the three groups, with age being a critical factor influencing pregnancy outcomes in IVF treatment. However, despite the obese group being younger on average than the normal weight group, pregnancy outcomes remained poorer. Notably, to minimize bias, we specifically selected women undergoing the antagonist protocol for their first cycle and adjusted potential confounders including age. Thus, our results were considered robust. Additionally, the homogeneity of our study population may limit the generalizability of our results to other ethnicities. Furthermore, we were unable to stratify PCOS into specific phenotypes due to unavailable information in the clinical database. Investigating the effect of obesity on different PCOS phenotypes should be a focus of future research.

In conclusion, our data indicate that among women with PCOS undergoing IVF-ET, overweight or obese cohorts exhibited lower live birth rates and higher rates of miscarriage in comparison to those with normal weight. Furthermore, overweight or obese individuals experienced prolonged stimulation periods and higher gonadotropin consumption, yielding fewer retrieved oocytes, reduced numbers of good quality embryos, and inferior pregnancy outcomes. The findings of our study provide valuable insights for physicians to offer more precise and personalized counseling to women with PCOS seeking IVF treatment. Such information holds significance for clinicians as it informs decision-making regarding IVF treatment strategies for women with PCOS.

## Data Availability

The datasets used for this study are available from the corresponding author on reasonable request.
